# Complexity beyond the usual suspects

**DOI:** 10.1007/s00787-025-02835-1

**Published:** 2025-08-07

**Authors:** Julian Koenig

**Affiliations:** Faculty of Medicine, Department of Child and Adolescent Psychiatry, Psychosomatics and Psychotherapy, University of Cologne, University Hospital Cologne, Cologne, Germany

This month’s issue of *European Child and Adolescent Psychiatry* posed considerable challenge to writing the editorial - covering the sheer breadth of topics addressed. Several sets of papers emerged early on, including collections of studies in this month’s issue, addressing attention deficit hyperactivity disorder (ADHD) or autism spectrum disorder. All of which deserve praise in their own regard. However, in reading yet another fine issue of our journal, articles addressing populations beyond the usual suspects sat with me. Not only do these studies attract our attention to vulnerable populations at the sidelines of child and adolescent psychiatry, but illustrate the complexity we are facing, in our joint endeavor to improve youth mental health.

In a case-control study, Shao et al. [1] investigated social and emotional outcomes of offspring born to mothers with systemic lupus erythematosus (SLE), as a potential consequence of inadequate intrauterine development. Children in the SLE group showed significant differences in birth-related outcomes (i.e., lower gestational age and size, birth weight, and length of gestation) and long-term physical development (i.e., greater incidence of obesity in the SLE group). Although motor and communication skills were largely unaffected, children born to mothers with SLE showed greater prevalence of abnormal social and emotional development. While the mechanisms underlying this association remain speculative, the work highlights the importance of considering trans-generational effects, and screening for developmental outcomes in children born to mothers with somatic conditions, potentially affecting long-term child development.

In a similar vein, the systematic review and meta-analysis by Damtie et al. [2], provides an update on the prevalence of ADHD in offspring of mothers with maternal diabetes. Independent of type (i.e., gestational diabetes or any pre-existing diabetes), analyses show robust evidence that children exposed to diabetes during prenatal development are at an increased risk of developing ADHD– suggesting a pooled 31% increased risk. In contrast to work in early stages on SLE, this fine meta-analysis included more than 18 million subjects, illustrating the necessity of continued research to derive robust conclusions. However, similar to SLE, still the exact mechanisms, linking maternal diabetes to offspring ADHD, are unknown. Sensitivity analyses by the authors, comparing pre-existing diabetes and gestational diabetes, suggest that longer exposure to and greater severity of the diabetic state, may be associated with greater ADHD incidence– providing some hints for future studies to follow-up on.

The mixed-method study by Loisel et al. [3] adds to the theme, addressing psychological distress in adolescents with obesity. The work is unique, in that it addresses not only adolescents’ self-reports but the clinicians’ perspective on the challenge in screening for mental health problems in this population. Results from the study suggest, that psychiatric conditions are not adequately addressed in adolescents with obesity– terming it as “gray area”. In context of the two prior studies and the current attention that glucagon-like peptide 1 receptor agonists receive, also in the treatment of psychiatric conditions [4], I echo the authors’ call to implement screening for psychiatric symptoms in adolescents presenting with obesity. It needs to be well understood, that respective symptoms (i.e., depression and anxiety) are not secondary to obesity but originate from joint biological mechanisms, initiating reciprocal reinforcement between behavior and emotional states.

Proper physical activity, reduced screen time, and good sleep are well-known as protective measures to prevent depressive symptoms in adolescents. While these likely tap into similar links of somatic and mental health, López-Gil et al. [5] present intriguing analyses from the Youth Risk Behavior Surveys, including more than 45,000 adolescents from the US - questioning a simplistic view. Although their analyses support the general assumption that adolescents adhering to respective 24-h movement recommendations show lowest risk for the development of depression, they caution us to assume that these principles are universal. Most interestingly, the authors illustrate that protective effects vary across sociodemographic groups. Notably, younger adolescents, females, and white individuals experienced greatest benefits. Further, effects showed heterogeneity over time, with largest effects in 2019 and 2021, suggesting potential links to environmental factors, including the COVID-19 pandemic. Preventive measures thus need to be tailored to specific sociodemographic groups and their effects need to be understood in interaction with current events, cautioning us to assume generalizability.

Like no other medical discipline, child and adolescent psychiatry deals with a multiverse of factors contributing to the etiopathology of mental ill-health, its prevention and treatment. Trans-generational effects, somatic comorbidity, and sociocultural specificity in an ever changing global context are just a few of the phenomena that the present issue highlights. As editors of *European Child and Adolescent Psychiatry*, we are committed to provide guidance for our readership by selecting only the best evidence to be published in our journal. It is inevitable that most of the times we are still left with more questions than answers. Nevertheless, I hope you enjoy the present issue as much as I did and hope you’ll have time to reflect and unwind during this summer.
